# A Mechanistic Model of Human Recall of Social Network Structure and Relationship Affect

**DOI:** 10.1038/s41598-017-17385-z

**Published:** 2017-12-07

**Authors:** Elisa Omodei, Matthew E. Brashears, Alex Arenas

**Affiliations:** 1 Departament d’Enginyeria Informàtica i Matemàtiques, Universitat Rovira i Virgili, 43007 Tarragona, Spain; 2 Department of Sociology, University of South Carolina. Sloan College Rm. 321, 911 Pickens St, Columbia, SC 29208 USA

## Abstract

The social brain hypothesis argues that the need to deal with social challenges was key to our evolution of high intelligence. Research with non-human primates as well as experimental and fMRI studies in humans produce results consistent with this claim, leading to an estimate that human primary groups should consist of roughly 150 individuals. Gaps between this prediction and empirical observations can be partially accounted for using “compression heuristics”, or schemata that simplify the encoding and recall of social information. However, little is known about the specific algorithmic processes used by humans to store and recall social information. We describe a mechanistic model of human network recall and demonstrate its sufficiency for capturing human recall behavior observed in experimental contexts. We find that human recall is predicated on accurate recall of a small number of high degree network nodes and the application of heuristics for both structural and affective information. This provides new insight into human memory, social network evolution, and demonstrates a novel approach to uncovering human cognitive operations.

## Introduction

The “Social Brain Hypothesis,” argues that human intelligence evolved to deal with social rather than physical challenges^1^. Studies of brain size and morphology support this argument^2–4^ and yield the prediction that human primary groups should contain roughly 150 individuals^5,6^ (i.e., “Dunbar’s Number”). This estimate has received empirical support^7,8^, but may underestimate human primary social group size^9,10^. Recent work helps to resolve this discrepancy by showing that humans use schemata (i.e., preexisting frameworks for understanding information that allow individuals to organize the learning experience and complete it more rapidly) as “compression heuristics”^11^ to facilitate the encoding and recall of social networks. These schemata allow individuals to discard most of the information on connections between individuals in favor of a heuristic for reconstructing the network from partial recall, allowing the compression of social information into a smaller memory space. Prior research has identified several compression heuristics, including triadic closure^12,13^ (e.g., if A <−> B and B <−> C then A <−> C), kinship^12,14^ (i.e., whether, and what type, of kin two individuals are), and affective balance^14^ (i.e., consistency with liked associates in affective judgments of third parties). These compression heuristics allow larger networks to be accommodated without concordant increases in brain mass. Related research shows that memory for social networks is fundamentally different than memory for identically structured, but non-social, networks, demonstrating the need to study social memory in particular^15,16^. Compression heuristics have also been shown to influence moral dilemma decision time^17^, and are consistent with fMRI studies showing differential recruitment of brain regions in the processing of kin and non-kin social ties^18^. We build on this research by using a mechanistic model to investigate the algorithmic processes employed by the brain to recall social information. Here we show that human network memory is consistent with a focus on accurate recall of a small number of high degree network nodes augmented by the application of heuristics for both structural and affective information.

The brain is an information-processing organ responsible for interpreting stimuli and generating responses, both automatically and under conscious control. Brain imaging studies provide indications of where brain activity occurs, but fail to indicate what operations the brain is performing. By analogy, using instrumentation to study the patterns of activation in a digital computer would allow researchers to localize where and when components were active, but would not reveal the operations performed therein. Social network encoding and recall depends on the algorithmic behavior of the brain, which therefore influences human behavior at the individual and aggregate levels. Recent fMRI research exhibits a sensitivity to this information processing logic, finding that the brain may run a number of social “daemons”, or automatic background processes that make social information cognitively available^19^. Prior attempts to explore social algorithms revealed that triads appear to be the default unit of network encoding^20^. We extend this research by constructing a mechanistic model of human network recall. If such a model is capable of producing simulated recall data that is consistent with observed data derived from laboratory studies then we will have demonstrated a sufficient set of operations to account for the brain’s observed behavior. This does not guarantee that the brain actually does execute this precise set of operations, but nonetheless allows parsimonious duplication of the brain’s observed behavior, thereby contributing to eventual computational models of human cognition. This research therefore provides new insight into human memory, social network evolution, and demonstrates a novel approach to uncovering human cognitive operations.

## Methods and Materials

We reanalyze data derived from an earlier experiment^14^. In this prior study 295 participants (186 female, 109 male) completed a 2 × 2 design intended to measure their ability to recall networks distinguished by their degree of affective balance^21,22^ and use of kin versus non-kin terminology. In a balanced network individuals share affective views of third parties with their positively tied associates (i.e., like those whom their friends like and dislike those whom their friends dislike), while imbalanced networks deviate from this pattern. Kin networks are described using kin labels for relationships (e.g., brother/sister) while non-kin networks are not (e.g., friend, coworker). All target networks were described in vignettes (i.e., short paragraphs of text presented to participants on a computer screen) without any visual aids. Artificial vignettes were used for three reasons. First, by constructing a vignette from scratch rather than relying on measurements of existing social networks we can determine exactly what the true state of the network is. This is essential for an investigation of memory. Second, vignettes allow the relationships to be presented in context (i.e., all ties presented together) whereas alternatives such as the paired-associates learning task present dyads in isolation. While it may appear to be unusual in real life to need to learn an entirely new network, it resembles the experience of entering a new social context (e.g., new job or club), which contains a set of existing relationships that must be learned as quickly as possible. Similarly, many forms of popular entertainment (e.g., television programs, movies, novels, etc.) require consumers to learn and track the relations between third-parties who are not known personally. As such, we expect most participants to be practiced at this task. Third, humans routinely exchange social information linguistically (e.g., gossip) and thus should be comfortable with this manner of presentation, thereby avoiding biases stemming from unfamiliarity. All characters and dyads were presented in the same order in all conditions, and none of the vignettes contained any plot or story, instead simply describing the relationships between the characters (e.g., “Henry is Alyssa’s brother, and they like each other, Henry is also Elizabeth’s son…”). The absence of narrative distinguishes these vignettes from gossip in more natural contexts that likely often contains a plot. However, this is necessary in order to avoid possible confounds stemming from variation between conditions in how interesting or entertaining the narratives are perceived to be. Narratives that are perceived to be more interesting are, ceteris paribus, likely to be recalled more accurately. By eliminating narrative entirely this potential confound is avoided. All conditions contained 15 characters and 23 undirected ties, while no condition contained directed ties, yielding a network density (i.e., number of observed ties divided by number of ties mathematically possible) of 0.219. The network size of 15 was chosen with the intention of stressing the participants; the number of individuals depicted is roughly double the estimated maximum capacity of working memory and the potential number of undirected relations (i.e., 105) is more than an order of magnitude greater. All vignettes contained two disconnected components (i.e., sub-groups with no connections between them), and the components did not vary in size by condition. The kin schema manipulation only impacted the terms used to describe the network and did not impact its structure. Both the balanced and imbalanced conditions consist of 15 undirected ties containing positive affect and 8 undirected ties containing negative affect. Thus, all conditions have the same number of positive and negative ties and the balance manipulation only alters whether these are configured into balanced or imbalanced structures. Informed consent was obtained from all research subjects, all relevant regulations and guidelines concerning the protection of human subjects were followed, and all procedures were approved by the Cornell University Institutional Review Board.

Participants began the experiment by sitting at a computer terminal and answering a series of simple demographic questions. The computer selected a vignette at random (one per participant) and presented it on screen as a paragraph of text. The participants were instructed to commit the information contained in the vignette to memory. Participants had unlimited time to study the vignette and were allowed to take notes on provided sheets of paper, but knew that the notes would be confiscated before the recall phase. The amount of time spent studying the vignette was measured without the participants’ knowledge. The experimenter was blind to the participant’s experimental condition.

The participant then completed a word span exercise^23^ with the experimenter, the task serving both as a standard measure of working memory, and as a means of clearing the participants’ working and sensory (i.e., auditory and visual) memory stores. In this exercise, the participant read a series of sentence sets out loud and, at pre-determined times, recalled the last word in each preceding sentence in the current set. The number of sentences per set gradually increased from a low of two to a high of seven, ending when the participant is either unable to correctly recall the final words for two out of three sets of a given size or obtained the maximum score. The sentences were drawn from popular press books, ensuring consistent readability, and contained between 13 and 16 words. At the end of the word span task the experimenter entered the participant’s score into the computer terminal, which the participant then used to complete the recall phase.

In the recall phase participants checked a series of boxes to indicate which characters had relationships with each other. On a second screen the participants indicated the valence (i.e., like vs. dislike) of each recalled relationship. Participants could return to the first screen of the recall phase and change their relationship selections as often as desired, but this cleared the relationship valence choices, and participants received no feedback on their answers. Participants supplied all of their judgments about relationship existence (i.e., anywhere from a total absence of any ties to a fully saturated network containing all 105 possible ties) for all characters, and affect for all characters they recalled as connected, on these two screens. Once participants advanced beyond the valence screen they could not return. Finally, participants were compensated and debriefed. All participants were told that the amount of compensation they would receive for completing the study was contingent on their success at recalling the vignette, but in fact all participants were compensated equally. The deception ensured that the participants were motivated to recall the information accurately. The experiment typically required forty-five to fifty minutes to complete, and all participants completed it.

In the current paper we make use of the 295 recalled networks, one for each participant, contained within the data generated by the original study. We use these recalled networks as comparison data for evaluating whether our mechanistic model produces networks similar to those generated by real human participants These data and other research materials are available from the corresponding author upon reasonable request.

## Results

**Figure 1:**
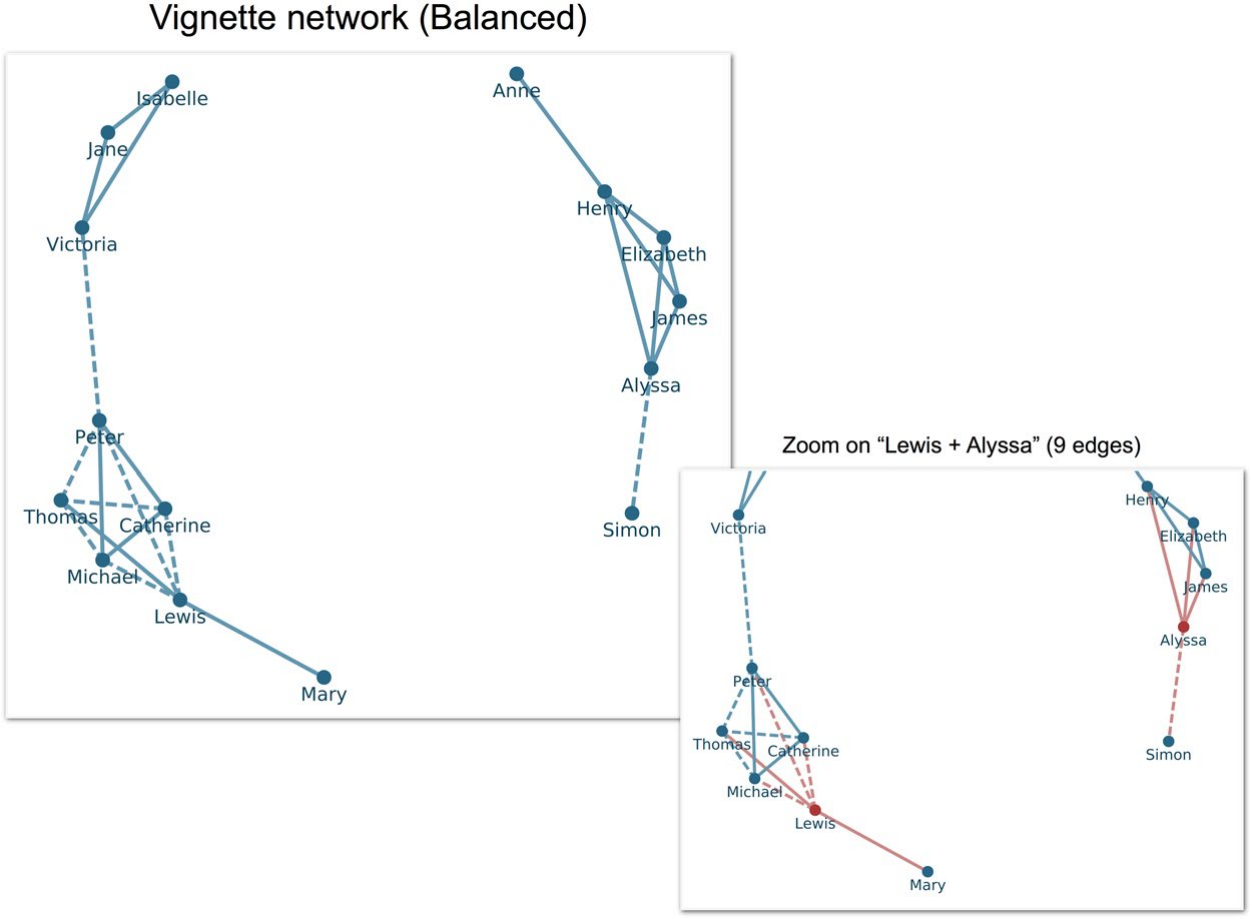
Sociogram of stimulus network presented as text to participants in the Balanced-Family condition. Solid ties are positively valenced (liking) while dashed lines are negatively valenced (disliking). Inset highlights ties incident to characters Lewis and Alyssa in red.

Prior research shows that network structure and dyadic affect are separable cognitive elements^14^ (i.e., recall of the existence of a tie is distinct from recall of the affect characterizing a dyad), so we begin by modeling network structure only. Because all conditions shared a common structure (the “reducible” network^12^; See Fig. 1), it is not necessary to distinguish this process by condition. We assume that individuals are able to recall all characters given in the vignette, which is reasonable given the size of our target networks as well as the limited number of ongoing close contacts maintained by individuals. First, the model selects all ties incident with one or more pre-selected individuals for recall. This provides an initial, non-random set of recalled dyads. Next, the model considers all pairs of currently unconnected characters, one pair per iteration. If adding a tie between a pair of characters would complete a triad, then it is added with a probability, ${P}_{Triangle}=\frac{{T}_{Closed}}{T}$, or the number of closed triplets in the target network (*T*_*Closed*_) divided by the total number of connected triplets in the target network (*T*). For example, in Fig. 1 <Anne, Henry and Elizabeth> and <James, Henry and Elizabeth> are both connected triplets, but only the latter is a closed connected triplet, because all three members of that triplet are connected to each other. In contrast, in the former triplet Anne is not connected to Elizabeth and therefore the triangle is not closed. If the tie is not part of a connected triplet, the tie is added with a probability, $P=\frac{|E|-{T}_{Closed}}{\frac{n(n-1)}{2}T}$, where *n* is the number of nodes in the target network and |*E*| is the number of edges in the target network. Adoption of *T*_*Closed*_ reflects the assumption that individuals perceive reasonably accurately how much closure is present within a network. As such, we should expect the participants to use this overall level of closure as a clue in deciding whether to close an incomplete triad. Likewise, the probability of adding a tie that is not part of a connected triplet *P* is based primarily on the assumption that participants recall the approximate density of the network. Denser networks should make participants more likely to infer that a given dyad contains a tie rather than is null. In both cases we use the true values from the target networks (i.e., those presented to the participants) under the assumption that these are likely to capture the central tendency of participant assessments. Obviously, if these assumptions are not reasonably valid, then our model should prove quite poor at capturing the observed recall behavior of the participants. The model is executed for various combinations of initially recalled ties incident to particular characters. Table 1 gives the number of ties initially recalled for each combination of characters tested. Given that this is a stochastic model, estimation is repeated 1,000 times for each set of initial ties (i.e., the model is fully executed 1,000 times for each set of initial ties). Any particular model execution is complete when all pairs of characters have been considered. Model performance is assessed using *precision*, or the number of correctly recalled ties divided by the number of recalled ties, and *coverage*, or the number of correctly recalled ties divided by the number of ties present in the target network. Comparisons between model results and experimental results rely upon the metric ${\rm{\delta }}=|{\overline{x}}_{{\rm{model}}}-{\overline{x}}_{\exp }|/{\sigma }_{\exp }\,$, where ${\overline{x}}_{{\rm{model}}}$ is the mean value of either precision or coverage as generated by the model and ${\overline{x}}_{\exp }$ is the corresponding value generated by experiment. σ_exp_ is simply the standard deviation for precision or coverage as generated by experiment. If *δ* < 1, the mean of the model is within less than a standard deviation of the experimentally derived results.

**Table 1:** List of tested values of initially recalled ties. The first column indicates the characters(s) whose ties are correctly recalled in the initial phase, and the second column reports the total number of ties incident to the named characters.

Characters	Number of ties
Isabelle	2
Victoria	3
James	3
Alyssa	4
Catherine	4
Lewis	5
Peter	5
James + Isabelle	5
Alyssa + Isabelle	6
James + Victoria	6
Catherine + Isabelle	6
Peter + Isabelle	7
Alyssa + Victoria	7
Catherine + Victoria	7
Lewis + Isabelle	7
Catherine + James	7
Lewis + Victoria	8
Peter + Victoria	8
Lewis + James	8
Peter + James	8
Catherine + Alyssa	8
Lewis + Alyssa	9
Peter + Alyssa	9
Catherine+James+Isabelle	9
Peter+James+Isabelle	10
Catherine+James+Victoria	10
Lewis+James+Isabelle	10
Catherine+Alyssa+Isabelle	10
Peter+Alyssa+Isabelle	11
Lewis+James+Victoria	11
Lewis+Alyssa+Isabelle	11
Peter+James+Victoria	11
Catherine+Alyssa+Victoria	11
Lewis+Alyssa+Victoria	12
Peter+Alyssa+Victoria	12

**Figure 2:**
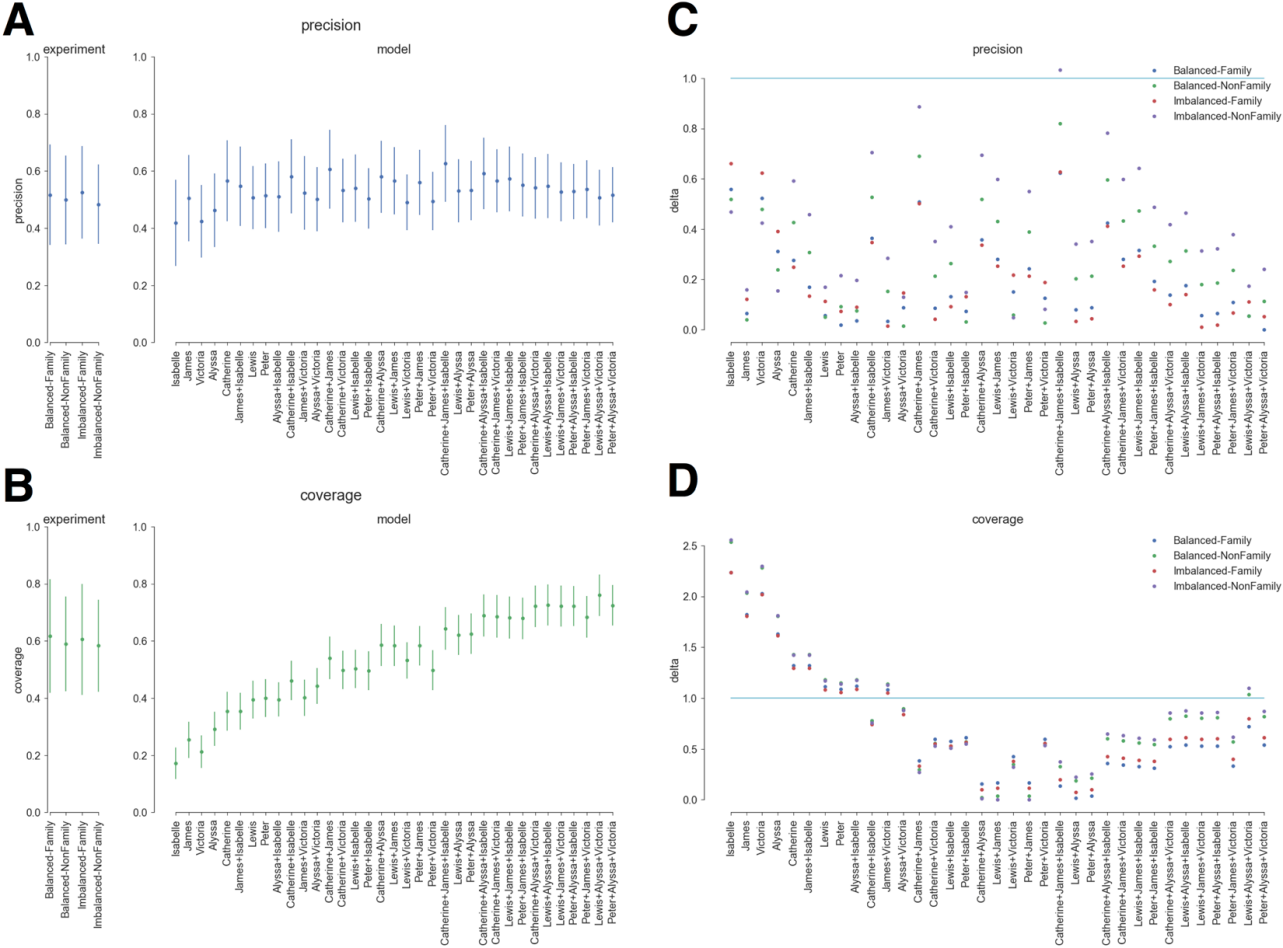
Panels A and B provide experimentally derived values (left most pane) for precision (Panel A) and coverage (Panel B). Right hand sections of Panels A and B provide precision and coverage, respectively, results for models based on ties incident to characters given in the corresponding column. Panels C and D provide *δ* values for precision and coverage, respectively.

Figure 2 depicts the results for precision (Panel A) and coverage (Panel B) as a function of different combinations of initially selected ties, as well as the corresponding *δ* values for each (Panels C & D, respectively). The left most section of Panels A and B give the experimentally derived values, and standard errors, for precision and coverage, respectively. The right hand sections of Panels A and B provide the values produced by models using initially recalled ties incident to the characters listed in the column label. The best results for both precision and coverage are obtained when 8 to 9 ties are recalled initially, incident to one high-degree character in each component (e.g., Lewis + Alyssa). This is consistent both with estimates that human working memory can accommodate roughly seven discrete pieces of information at a time^24,25^, and with the finding that many networks, social and non-social, are scale-free^26^. In such networks, initially remembering edges incident to high-degree nodes would be an excellent strategy for recalling large portions of the network structure.

As a robustness check, we fixed the set of initial ties to those incident to Lewis + Alyssa (See Fig. 1 inset) and varied the values of *P*_*Triangle*_ and *P* by altering the value of *T*_*Closed*_ to capture the possibility that individuals may mis-remember the triadic density (the value of *P* is impacted by this approach because it captures the density of the non-triadic portions of the target network). We selected Lewis+Alyssa because each is a high degree character in their own component and because Welch’s *t*-test shows that the mean of the model results for precision, coverage, and quality does not significantly differ from experimentally derived values in any but one (Imbalanced-Non-Kin precision) case. The results (Figure S1) show that coverage (Panel B) is relatively insensitive to mis-recall of *T*_*Closed*_ while precision (Panel A) is more impacted, though in virtually all cases the experimentally-derived values remain within a standard error of model predictions. Likewise, the *δ* metric shows that in virtually all cases for precision (Panel C) the model is within a standard deviation of experimentally derived results, while in all cases modeled coverage values are within a standard deviation of experimentally derived values (Panel D). Our approach therefore appears to capture observed recall of network structure accurately.

Next, we consider the valence of the ties, determining whether they are positive (i.e., liking) or negative (i.e., disliking). We begin by assuming that the valences of initially recalled ties are also recalled correctly. This is equivalent to assuming that the process of reconstructing the affective makeup of a network begins with a small number of ties about which the individual is certain, much as recall of network structure begins with a handful of correctly recalled ties. Next, new ties are created in the manner given above. If the new tie completes a connected triplet then it will be assigned a positive valence if the sign product of the open triplet is positive, and a negative valence if the sign product of the open triplet is negative. In the event that the tie closes multiple triplets, the valence is assigned based on the average of the sign products of the open triplets, with a positive average yielding a positive valence and a negative average yielding a negative valence. This rule ensures that recalled valences maximize triadic balance in accordance with established research^27,28^ as well as the finding that balance appears to be a default heuristic^14^. In the event that the new tie does not close a connected triplet, the tie is assigned a positive valence in accordance with the positivity bias described in prior studies^29^. Model fit is assessed using *quality*, defined as the number of correctly recalled valences divided by the number of correctly recalled ties.

**Figure 3:**
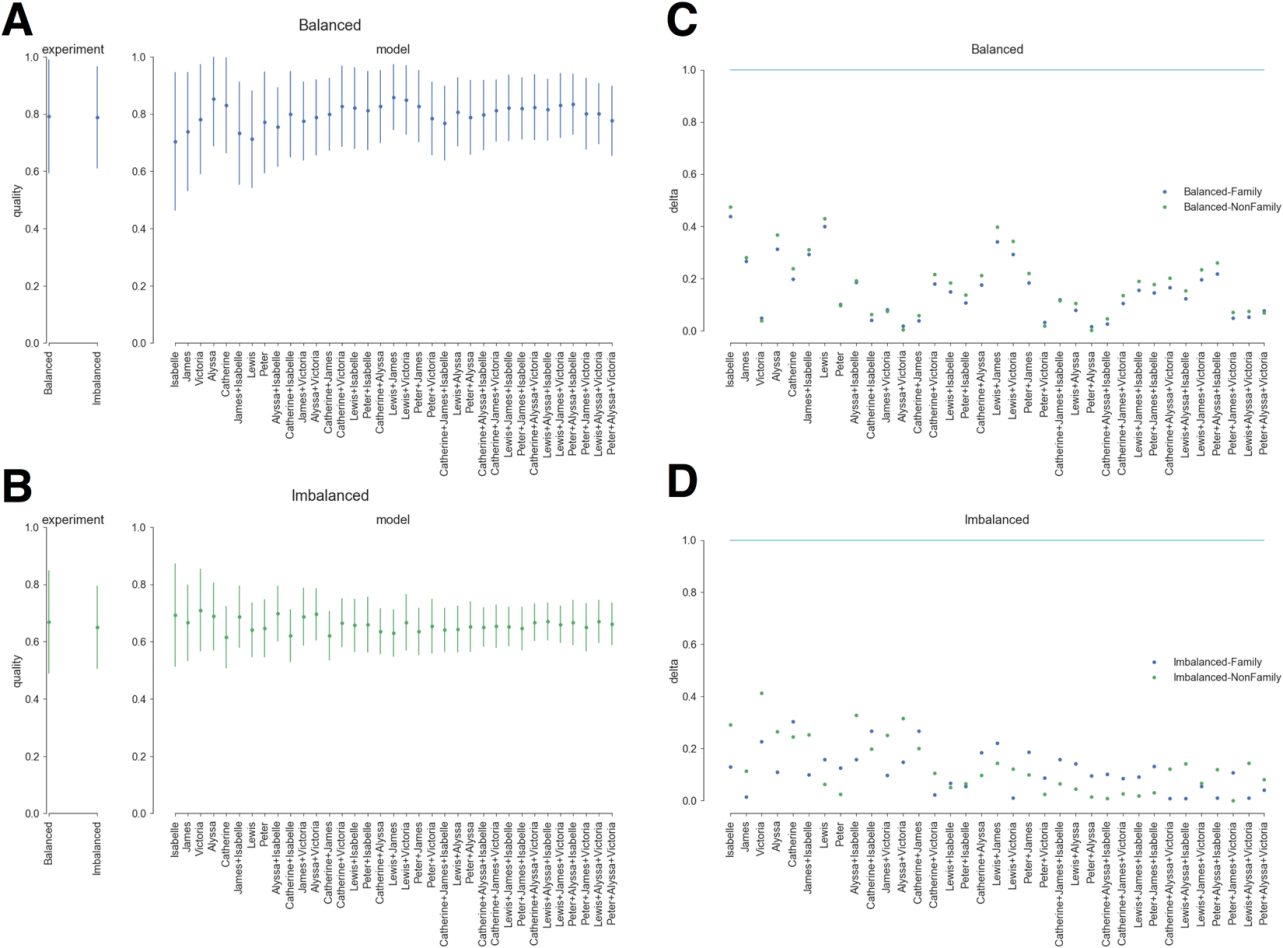
Panels A and B provide experimentally derived values (left most pane) for balanced (Panel A) and imbalanced (Panel B) conditions. Right hand sections of Panels A and B provide quality results for balanced and imbalanced conditions, respectively, for models based on ties incident to characters given in the corresponding column at *x* = 30%. Panels C and D provide *δ* values.

Results indicate that in the imbalanced conditions this procedure reproduces the experimental results quite accurately, but in the balanced conditions it accurately reproduces the target network while significantly overestimating the quality obtained in the experimentally derived networks. To correct this issue we assume that a certain proportion, *x*, of the initially recalled ties are remembered with randomly-chosen valences. We find (Fig. 3) that a value of *x* = 30% produces results that are a good fit to experimentally determined values in the balanced conditions (Panel A), and yields essentially unchanged model results for the imbalanced conditions (Panel B). Corresponding *δ* results for the balanced (Panel C) and imbalanced (Panel D) conditions shows model quality results are solidly within a one standard deviation window of experimentally derived values. In a series of robustness checks we fixed the initially recalled ties to those incident to Lewis + Alyssa and varied the value of *x*. Results show that the model performs well for the imbalanced conditions across all values of *x* (Figure S2, Panels B and D), while fitting best for the balanced conditions when *x* = 20–45% (Figure S2, Panels A and C).

## Discussion

The mechanistic model described above produces networks that strongly resemble the experimental results. This does not guarantee that human brains are actually executing identical procedures in recalling social networks, but shows that these operations are sufficient to reproduce the observed outcomes. However, there are several indications that our mechanistic model is capturing the decision behavior of the human brain. First, our model works best with an initially recalled number of ties that is consistent with the empirically estimated capacity of working memory. While individuals can doubtless remember more total ties in long-term memory than this, it suggests that when loading a network into working memory individuals add directly recalled ties until they experience memory pressure, and then rely on heuristics to supply the rest. This in turn implies that heuristics may be primarily useful when recovering networks from memory, rather than in encoding them initially.

Second, the model achieves the best fit to data when the initially recalled edges are incident to a highly connected individual in each component of the target network. This suggests that human memory may be biased in favor of recalling the associates of high degree individuals (hubs). Or, more directly, our recall tends to be focused on those who are most popular^30^. Not only is this consistent with the well-known tendency for individuals to value connections to popular others, but it is consistent with the finding that many social networks present a power law degree distribution. In such networks, relatively small numbers of nodes have large numbers of connections, and so recall focused on these high degree individuals would be quite successful. This finding is especially interesting given that the original target networks were not designed to be power law distributed and do not exhibit this tendency, and so its emergence here is not simply a demand characteristic of the experiment.

Third, our initial model for dyadic valence was accurate for imbalanced networks, but far more accurate than experimentally derived values for the balanced networks. Subsequent modifications showed that if slightly less than one initial dyad out of three was remembered with a random valence, model prediction fit experimental results quite well. This is consistent with humans who have imperfect, though still good, direct memory of dyadic affect but who nonetheless reliably follow a set of heuristics in reproducing the network. In short, while we cannot demonstrate that the human brain actually uses these specific operations, its behavior appears to be consistent with these specific operations. Even if our model does not precisely replicate the algorithms used by the human brain, it does provide a parsimonious way of generating predictions for what should be recalled on average. Deviations from these predictions can be used to further refine the model, eventually contributing to a computational model of human social cognition. Such a model would permit understanding of how the brain calculates social behavior, helping to bridge the gap between brain localization and behavior. However, the current mechanistic model represents only a small part in that larger project and should not be expected to apply equally well to all possible social memory tasks. Our model was constructed using data from a laboratory experiment, and thus is an oversimplification of more natural recall processes. Social interaction in “the wild” includes more, and more complex, input and therefore our model should be viewed as only a first step.

Our results show that the amount of variance in outcomes for *precision*, *coverage*, and *quality* seems to be considerably greater in the model results than between experimental condition averages. This quite sensibly indicates that the success of compression heuristics is heavily dependent on the initial set of seeds that those heuristics reconstruct the network from. If humans systematically rely upon recall of high-degree individuals when reconstructing the network, we should anticipate that the experimental results will be more tightly clustered simply because the model explores a wider swath of the parameter space. By uncovering several of the algorithmic processes used to recall social networks this research both adds to our understanding of network cognition, and helps uncover the algorithms at work in the human mind.

## Electronic supplementary material

Supplementary Analyses
