# Supplementary material for: A Mechanistic Model of Human Recall of Social Network Structure and Relationship Affect
Source: Sci Rep. 2017 Dec 7;7:17133. doi: 10.1038/s41598-017-17385-z (PMC5719413; doi:10.1038/s41598-017-17385-z)
Supplement: Supplementary file 1 — Supplementary Analyses [file 41598_2017_17385_MOESM1_ESM.doc]

A Mechanistic Model of Human Recall of Social Network Structure and Relationship Affect

Elisa Omodei[[1]](#footnote-2)*

Matthew E. Brashears[[2]](#footnote-3)** (Corresponding Author)

Alex Arenas*

Keywords: Social Networks; Social Brain Hypothesis; Mechanistic Model; Cognition

Acknowledgements

MEB acknowledges financial support from the USA National Science Foundation (Award #1059282). AA acknowledges financial support from the ICREA Academia, the James S. McDonnell Foundation (Award #220020325), and MINECO (project FIS2015-71582-C2).

**Supplementary Information**

Figure S1- Recall precision (Panel A) and coverage (Panel B) derived from experiment (left hand side) and model (right hand side) when varying the number of recalled closed triplets. Panels C and D provide the corresponding
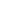
 values.


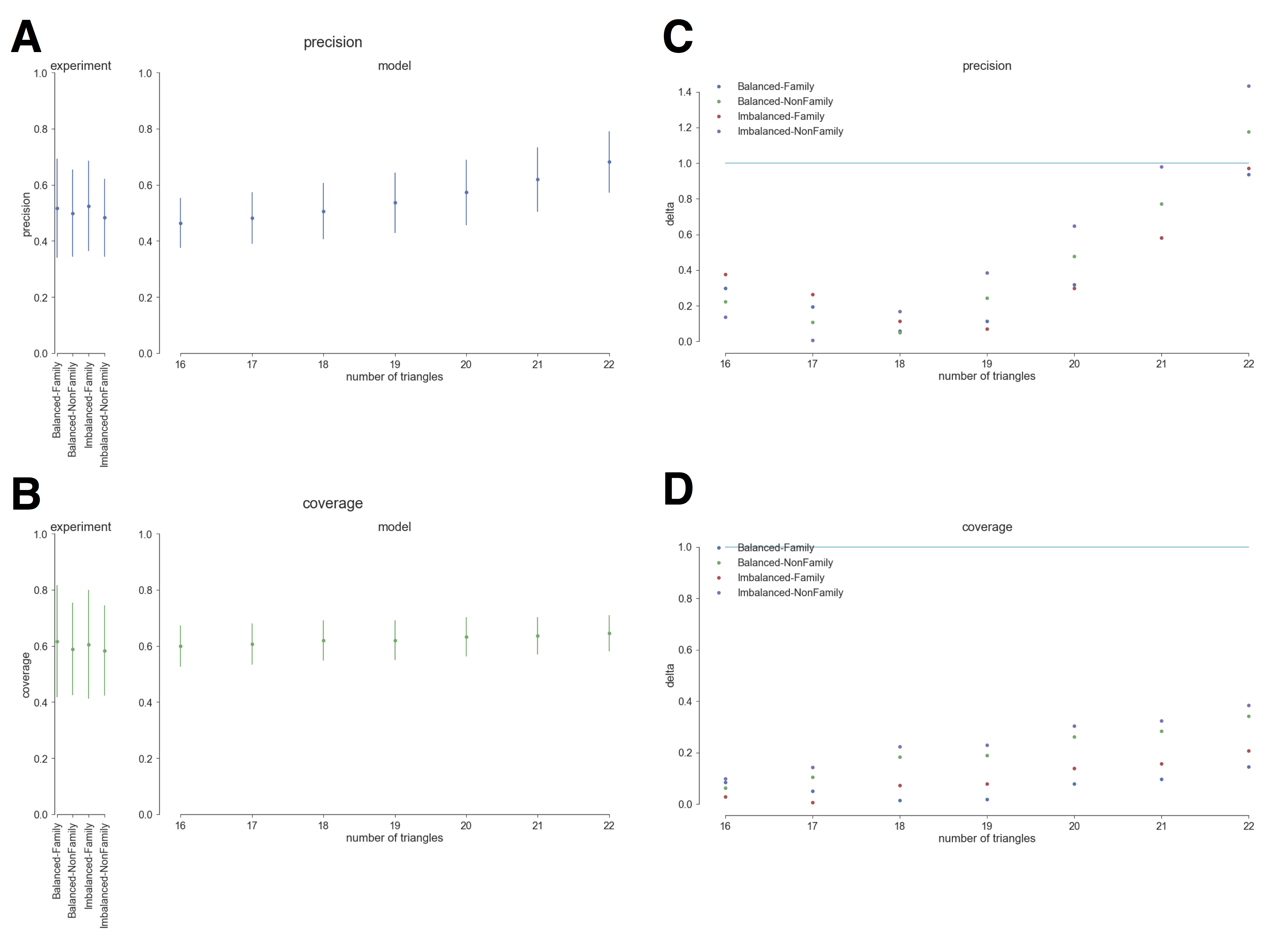


Figure S2- Recall quality in the balanced (Panel A) and imbalanced (Panel B) conditions when a proportion of initially recalled ties, *x*, are assigned a random valence. Model results include experimental means within one standard error at all values of *x* from 0% to 50%. Panels C and D provide the corresponding
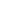
 values.


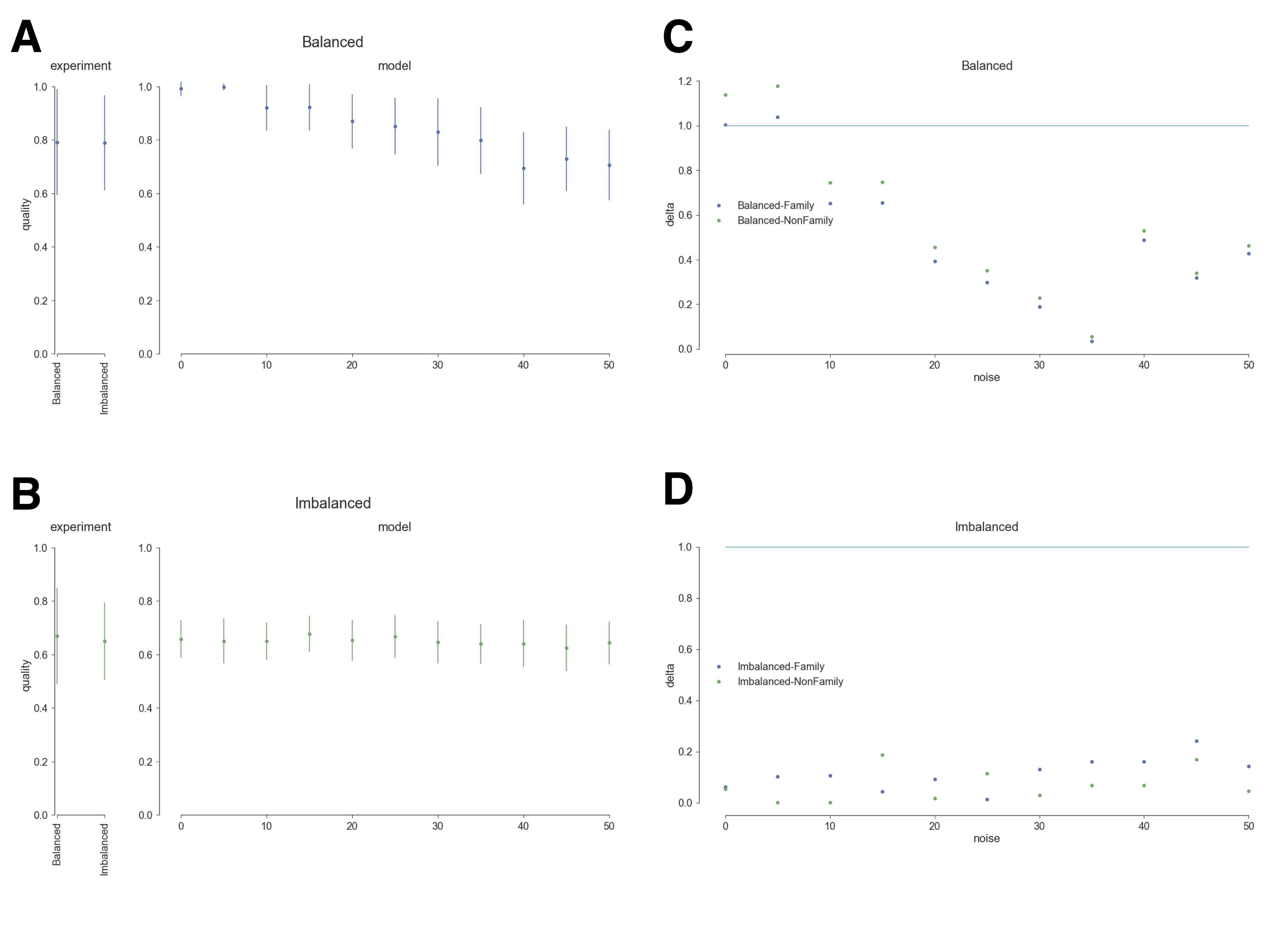


1. ^*^ Departament d’Enginyeria Informàtica i Matemàtiques, Universitat Rovira i Virgili, 43007 Tararagona, Spain [↑](#footnote-ref-2)
2. ^**^ Department of Sociology, University of South Carolina. Sloan College Rm. 321, 911 Pickens St. Columbia, SC 29208 [↑](#footnote-ref-3)
